# Postnatal growth and body composition in extremely low birth weight infants fed with individually adjusted fortified human milk: a cohort study

**DOI:** 10.1007/s00431-022-04775-3

**Published:** 2023-01-04

**Authors:** Tania Perrin, Pierre Pradat, Julie Larcade, Marion Masclef-Imbert, Blandine Pastor-Diez, Jean-Charles Picaud

**Affiliations:** 1 Service de Néonatologie, Hospices Civils de Lyon, Hôpital Universitaire de La Croix-Rousse, 103 Grande Rue de La Croix-Rousse, 69004, Lyon, 69677 France; 2 Centre de Recherche Clinique, Hospices Civils de Lyon, Hôpital Universitaire de La Croix-Rousse, Lyon, 69004 France; 3 Laboratoire CarMen, INSERM, INRA, Université Claude Bernard Lyon1, Pierre-Bénite, Lyon, 69310 France

**Keywords:** Nutrition, Prematurity, Breastfeeding, Fat mass, Fortification

## Abstract

This cohort study aimed to evaluate the impact of an individualised nutritional care approach combining standardised fortification with adjustable fortification on postnatal growth and body composition in extremely low birth weight (ELBW) infants. We included ELBW infants admitted to a neonatal intensive care unit and still hospitalised at 35 weeks postmenstrual age (PMA). The fortification of human milk was standardised (multicomponent fortifier) between 70 mL/kg/day and full enteral feeding, and then individualised using adjustable fortification. When weight gain was below 20 g/kg/day, protein or energy was added when serum urea was below or above 3.5 mmol/L, respectively. Postnatal growth failure (PNGF) was defined as being small for gestational age at discharge and/or when the *Z*-score loss between birth and discharge was higher than 1. Body composition was assessed between 35 and 41 weeks of PMA. Among the 310 ELBW infants included, the gestational age of birth was 26.7 ± 1.8 weeks, and the birth weight was 800 ± 128 g. The mean *Z*-score difference between birth and discharge was moderately negative for the weight (−0.32), more strongly negative for length (−1.21), and almost nil for head circumference (+ 0.03). Only 27% of infants presented PNGF. At discharge, fat mass was 19.8 ± 3.6%. Multivariable analysis showed that the proportion of preterm formula received and gestational age at birth were independently associated with the percentage of fat mass.

*  Conclusion*: The individualised nutritional care approach applied herein prevented postnatal weight loss in most infants, limited length growth deficit, and supported excellent head circumference growth.

**Table:** 

**What is Known:** *• At least half of extremely low birth weight infants are small for gestational age at discharge and postnatal growth deficit has been associated with impaired neurocognitive and renal development.* *• Human milk is the main milk used in neonatology and, although fortification of human milk is a standard of care, there is no consensus regarding the optimal fortification strategy to be adopted.*	
**What is New:** *• Using an approach combining standardised fortification followed by individualised adjustable fortification limited postnatal growth deficit for body weight and head circumference. Postnatal growth failure is not a fatality in extremely low birth weight infants.* *• Each additional gestational age week at birth resulted in a decrease in fat mass percentage at discharge, which was higher than in foetuses of the same gestational age, likely representing a necessary adaptation to extra-uterine life.*

**What is Known:**

*• At least half of extremely low birth weight infants are small for gestational age at discharge and postnatal growth deficit has been associated with impaired neurocognitive and renal development.*

*• Human milk is the main milk used in neonatology and, although fortification of human milk is a standard of care, there is no consensus regarding the optimal fortification strategy to be adopted.*

**What is New:**

*• Using an approach combining standardised fortification followed by individualised adjustable fortification limited postnatal growth deficit for body weight and head circumference. Postnatal growth failure is not a fatality in extremely low birth weight infants.*

*• Each additional gestational age week at birth resulted in a decrease in fat mass percentage at discharge, which was higher than in foetuses of the same gestational age, likely representing a necessary adaptation to extra-uterine life.*

## Introduction

Postnatal growth failure, which is associated with impaired neurocognitive and renal development [1–4], was observed in nearly 100% of very low birth weight infants at the end of the 1990s and still occurs in more than half of these infants [5]. In the Swedish EXPRESS cohort of ELBW infants, the median *Z*-scores for body weight were −0.66 at birth and −1.84 at 36 weeks PMA. Importantly, 44% of these infants had a *Z*-score below -2 standard deviation (SD) at discharge [6]. Although length growth deficit is also very common, the vast majority of children gradually catch up between the ages of 2 and 8 years and are within normal height ranges as adults [7, 8]. A deficit in fat-free mass (FFM) at discharge has been associated with suboptimal neurological outcomes, and the proportion of fat mass (FM) is known to be higher in premature infants compared to foetuses of the same gestational age (GA) [9, 10].

Human milk fortification strategies used in neonatal units worldwide are highly variable [11, 12]. Individualised fortification, whether adjustable or targeted, achieves better postnatal growth than standardised fortification [13, 14]. Adjustable fortification consists in modulating protein enrichment according to serum urea levels, while targeted fortification is based on the analysis of breast milk to adjust protein or energy content [11, 13]. The latter is time- and resource-consuming and its superiority over adjustable fortification have not been demonstrated [14].

The aim of this study was to evaluate the frequency of postnatal growth deficit and assess body composition at discharge in ELBW infants using an individualised nutritional care approach combining standardised fortification followed by adjustable fortification adapted to weight gain and serum urea.

## Population and methods

This single-centre retrospective observational study included infants born with a birth weight less than 1000 g, admitted within the first 24 h of life to the neonatal intensive care unit of the Croix-Rousse University Hospital in Lyon (France), and still hospitalised at 35 weeks PMA. Infants with serious congenital malformations were excluded.

Data were prospectively recorded in the patient’s electronic files (ICCA, Philips, Boblingen, Germany). Daily protein and energy intakes were assessed on the first day of each week of life and compared to recommended intakes (protein: < 1 kg: 4.0–4.5 g/kg/day, 1–1.8 kg: 3.5–4.0 g/kg/day, energy: 110–135 kcal/kg/day) [15]. Serum urea was measured weekly. Bronchopulmonary dysplasia (ventilatory support or oxygen therapy at 36 weeks PMA), intraventricular haemorrhage grade 3 or 4, periventricular leukomalacia, retinopathy of prematurity stage ≥ 3, and necrotising enterocolitis stage ≥ 2 were collected.

Body weight was measured daily during the first week of life, and then weight, crown-heel length, and head circumference (HC) were measured weekly. The length was measured using a rigid measuring board suitable for premature newborns (Premie Stadiometer, Ellard instrumentation, Monroe, USA). Anthropometric data were expressed in absolute values and *Z*-scores, and differences in *Z*-scores between birth and discharge were calculated [16]. Infants were considered to be small for GA (SGA) when the *Z*-score for body weight was ≤  −1.28 (10th percentile equivalent) [16]. PNGF was considered when the *Z*-score loss between birth and discharge was higher than 1 or when the Z-score for body weight at discharge was ≤  −1.28. Air displacement plethysmography (PEA POD®, Cosmed France, Brignais, France) was performed between 35 and 41 weeks PMA. Both FM% and absolute values of FFM were collected. Since at 35 to 41 weeks, infants were 2 to 4 months old, the data from infants born at 35–41 weeks as well as the data obtained in 2-month-old term infants were used as a reference [10].

Parenteral nutrition was started within the first 2 h of life, was individualised as soon as possible (within 48 h), and continued until the enteral ration reached 120 mL/kg/day. Enteral nutrition started on the first day of life using donor human milk. Then, the mother’s own milk was introduced as soon as possible, when available. It was pasteurised up to 32 weeks of corrected age [17]. Enteral nutrition increased daily from 15 to 20 mL/kg/day, up to 160 mL/kg/day. Energy supplement started at the end of parenteral nutrition and continued until milk intake reached 160 mL/kg/day: Liquigen® 4 g/100 mL (Nutricia, Saint Ouen, France). Fortification of human milk was started when enteral nutrition reached 70 mL/kg/day. Initially, all infants received a standardised fortification with a powder multicomponent fortifier: Fortipre® 4 g powder/100 mL (Nestlé, Noisiel, France) or Fortema® 3 g powder/100 mL + Nutriprem® 0.5 g powder/100 mL (Bledina-Danone, Limonest, France) (Table 1) [11]. Weight gain calculation and serum urea assessment were performed weekly. When weight gain was insufficient, i.e. < 20 g/kg/day, enteral intake was increased to 180 mL/kg/day. If it remained insufficient after 1 week, the fortification was individualised. Individualised adjustable fortification consisted of the addition of a protein supplement (Nutriprem® 1 g/100 mL) if serum urea was low (< 3.5 mmol/L) or an energy supplement (Liquigen®: 4 g/100 mL) if the serum urea was normal (> 3.5 mmol/L). Additional protein was reduced if serum urea was above 6.5 mmol/L. Fortification of human milk was maintained until body weight reached 1800 g. When the mother had not—or not enough—milk, donor human milk was used to complete or replace the mother’s own milk, and then, when body weight was equal to 1800 g, donor human milk was replaced by a preterm formula.

**Table 1:** Composition of multicomponent fortifiers and protein supplement (per 100 g powder)

	**MCF1**	**MCF2**	**Ps**
Energy, kcal	347	435	338
Protein, g	25.2	35.5	82.1
Carbohydrate, g	62.2	32	2.2
Sodium, mg	8	9.2	7.8
Calcium, mg	14.9	18.9	5.2
Phosphorus, mg	8.7	11	5.2

MCF1: Fortipre® (Nestlé, Noisiel, France), MCF2: Fortema® (Bledina-Danone, Limonest, France), Ps: Nutriprem® (Nutricia, Saint Ouen, France)

*MCF *multicomponent fortifier, *Ps *protein supplement

For statistical analysis, continuous variables were described by their means and SD, and comparisons were performed using Welch’s *t*-test or the nonparametric Mann–Whitney test. Categorical variables were described by the number of occurrences and percentages, and comparisons were performed using the chi-square test or Fisher’s exact test, as appropriate. Univariable and multivariable logistic regression analyses were performed to identify factors potentially associated with PNGF. Variables with *p* < 0.05 in univariable analysis were retained in the multivariable model. Results are presented as odds ratios and their 95% confidence intervals [95%CI]. The search for factors potentially associated with FM% was carried out using univariable and multivariable linear regression analyses. Variables with *p* < 0.05 in univariable analysis were retained in the multivariable model. Results are presented as an estimate with [95%CI]. The alpha-risk significance level for all analyses was set at 0.05. All analyses were performed using R software version 3.5.3 (R Foundation for Statistical Computing, Vienna, Austria).

The study was approved by the ethics committee Comité de Protection des Personnes Sud-Est IV (IRB: 00009118), and the institutional review board (Comité scientifique et éthique des Hospices Civils de Lyon, n°22_608) was registered in Clinicaltrials.gov (NCT02686801).

## Results

**Fig. 1:**
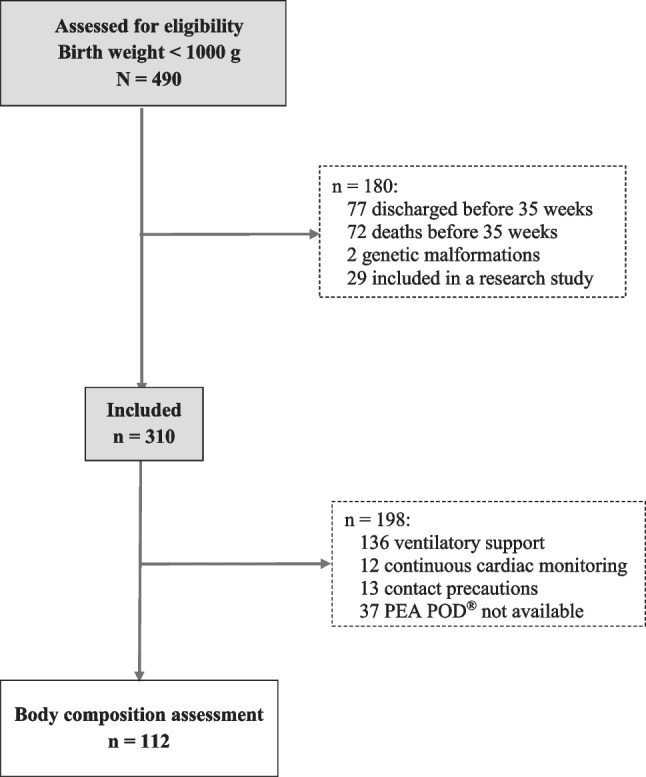
Flow chart

Between April 1, 2014, and December 31, 2019, 310 infants among the 490 infants with a birth weight less than 1000 g admitted to the neonatal intensive care unit were included. Body composition was assessed in 112/310 (36%) infants (Fig. 1). At birth, mean ± SD GA was 26.7 ± 1.8 weeks with a minimum of 23 weeks. The mean birth weight was 800 ± 128 g with a minimum of 440 g **(**Table 2).

**Table 2:** Characteristics of 310 extremely low birth weight infants at birth and during hospitalisation

**Characteristics**	**Total**
Antenatal steroids, *n* (%)	300 (97)
Gestational age at birth, weeks	26.7 (± 1.8)
Male sex,* n* (%)	140 (45)
Birth weight, g	800 (± 128)
Small for gestational age, *n* (%)	104 (33)
Bronchopulmonary dysplasia, *n* (%)	228 (74)
Postnatal steroids, *n* (%)	117 (37)
Periventricular leukomalacia, *n* (%)	5 (2)
Intraventricular haemorrhage grades 3 and 4, *n* (%)	16 (5)
Retinopathy of prematurity stage ≥ 3, *n* (%)	10 (3)
Necrotising enterocolitis ≥ 2,* n* (%)	4 (1.3)
Parenteral nutrition duration, days	19 (± 11)
Gestational age at discharge, weeks	38 (± 1.5)
Length of stay, days	79 (± 16)

Values are expressed as number (percentage) or mean (± 1 standard deviation)

**Fig. 2:**
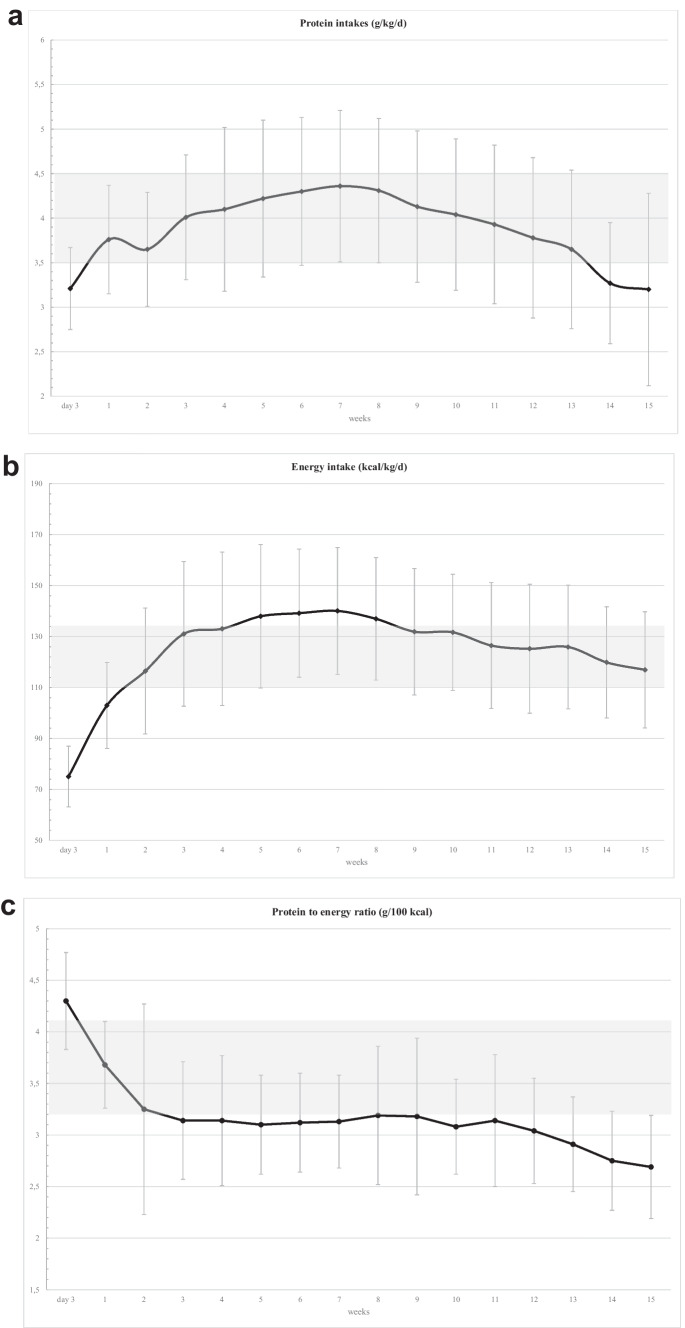
Weekly protein intake **a**, energy intake **b**, and protein to energy ratio **c **in 310 extremely low birth weight infants between birth and discharge, expressed as mean and standard deviation. Grey zones represent recommended intakes (Ref. 15)

Recommended protein intakes were reached before the end of the first week of life. Recommended energy intakes were reached at the end of the second week and were exceeded between the third and eighth week of life, bringing the protein-energy ratio slightly lower than recommended (Fig. 2). Mean serum urea levels were 4.2 ± 3.3 mmol/L at 1 month of life, 3.8 ± 2.4 mmol/L at 6 weeks of life, and 3.5 ± 1.9 mmol/L at discharge. The threshold value of 6.5 mmol/L was exceeded in 14.8% of infants at 1 month, 8.2% at 6 weeks, and 6.0% at discharge.

**Fig. 3:**
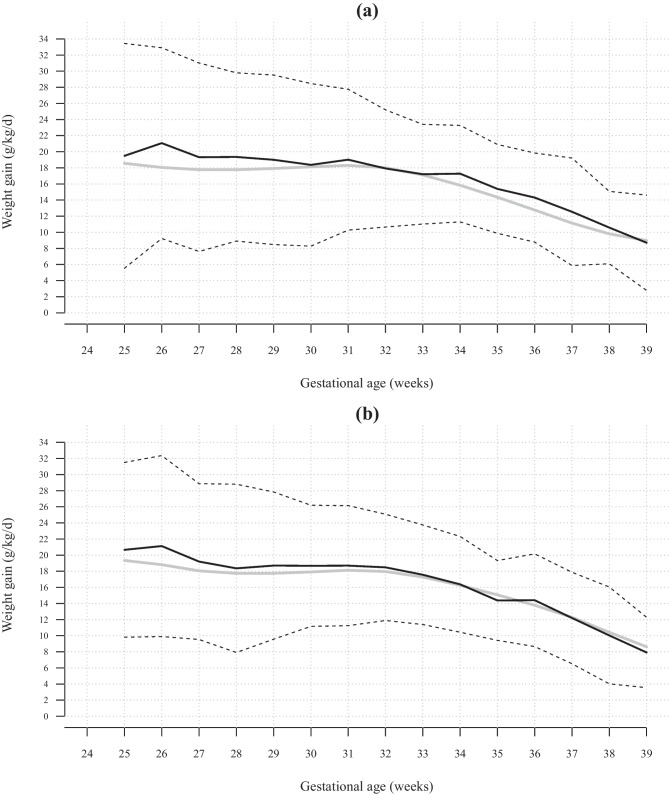
Postnatal weight gain (g/kg/day) in 310 extremely low birth weight infants (**a** boys; **b** girls). The grey lines represent the Fenton weight gain curves, and the black lines represent the mean weight gain of the present cohort (solid line) + / −1 standard deviation (dotted lines)

**Fig. 4:**
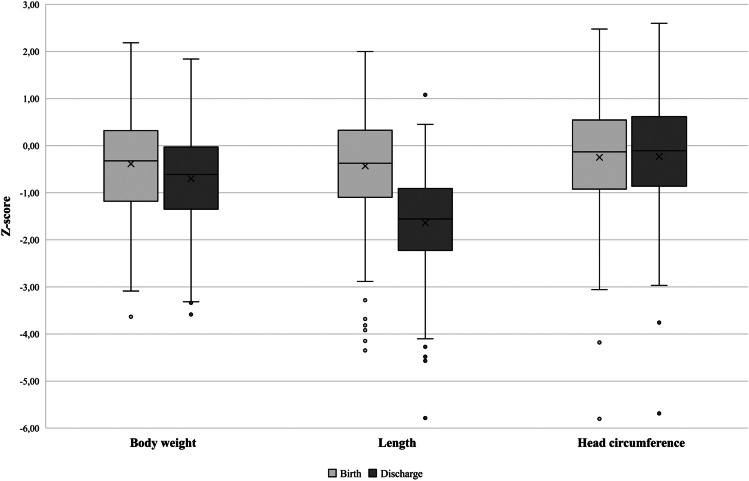
Differences in *Z*-score for body weight, crown-heel length, and head circumference between birth (light grey) and discharge (dark grey) in 310 extremely low birth weight infants. Expressed as boxplot (median, P25, P50, min, max)

At birth, the mean *Z*-score was −0.39 for body weight, −0.44 for length, and −0.26 for HC. The mean initial weight loss was 10 ± 4% of birth weight (Table 3). Thereafter, weight gain in both boys and girls closely followed the reference curves (Fig. 3). At 1 month of life, 62 infants (20.0%) had a body weight < 10th percentile. The mean *Z*-score difference between birth and discharge was almost nil for HC (0.03 ± 1.12), moderately negative for the weight (−0.32 ± 0.75), and more strongly negative for length (−1.21 ± 0.92) (Fig. 4). Overall, 84 infants (27.1%) had a body weight < 10th percentile and 26 (8.4%) had a *Z*-score for body weight below the 3rd percentile at discharge. A total of 114 infants (36.8%) presented PNGF (Table 3). The multivariable analysis identified SGA at birth and the use of postnatal steroids as independent risk factors for PNGF. The proportion of total milk intake as the preterm formula was a protective factor (Table 4).

**Table 3:** Anthropometric data and postnatal growth in 310 extremely low birth weight infants

**Body weight**	
Birth weight, g	800 (± 128)
*Z*-score birth weight	−0.39 (± 0.98)
Initial weight loss, % of birth weight	10 (± 4)
Weight at discharge, g	2790 (± 513)
Weight *Z*-score at discharge, SD	−0.71 (± 0.94)
Weight Δ*Z*-score between birth and discharge	−0.32 (± 0.75)
Loss in body weight *Z*-score from birth to discharge ≥ 1SD, *n* (%)	51 (17)
Weight at discharge < 10th percentile, *n* (%)	84 (27)
Postnatal growth failure* for body weight, *n* (%)	114 (37)
**Crown-heel length**	
Length at birth, cm	33.2 (± 2.2)
Length *Z*-score at birth, SD	−0.44 (± 1.15)
Length at discharge, cm	45.1 (± 2.7)
Length *Z*-score at discharge	−1.65 (± 1.02)
Length Δ*Z*-score between birth and discharge	−1.21 (± 0.92)
Loss in length *Z*-score from birth to discharge ≥ 1SD, *n* (%)	183 (59)
Length at discharge < 10th percentile, *n* (%)	188 (61)
Postnatal growth failure* for length, *n* (%)	246 (80)
**Head circumference**	
Head circumference at birth, cm	23.7 (± 1.5)
Head circumference *Z*-score at birth, SD	−0.26 (± 1.05)
Head circumference at discharge, cm	33.5 (± 1.8)
Head circumference *Z*-score at discharge, SD	−0.23 (± 1.12)
Head circumference Δ*Z*-score between discharge and birth, SD	0.03 (± 1.10)
Loss in head circumference *Z*-score from birth to discharge ≥ 1SD, *n* (%)	42 (14)
Head circumference at discharge < 10th percentile, *n* (%)	53 (17)
Postnatal growth failure* for head circumference, *n* (%)	76 (25)

^*^ Loss in *Z*-score for given anthropometric parameter, from birth to discharge, equal or higher than 1 standard deviation (SD) and/or *Z*-score for body weight at discharge less than −1.28 (10th percentile equivalent)

**Table 4:** Risk factors of postnatal growth failure in 310 extremely low birth weight infants

	**Univariable analysis**		**Multivariable analysis**	
**Risk factors**	**OR [95%CI]**	***p***	**OR [95%CI]**	***p***
Gestational age at birth, weeks	1.32 [1.15; 1.52]	< 0.001	/*	
Sex (female vs male)	1.03 [0.65; 1.63]	0.909	/	
Antenatal steroids	0.71 [0.19; 2.71]	0.620	/	
*Z*-score birth weight	0.59 [0.46; 0.77]	< 0.001	/	
Small for gestational age	5.12 [3.05; 8.57]	< 0.001	6.91 [3.80; 12.6]	< 0.001
Protein intake at day 7, 0.5 g/kg/day	0.56 [0.26; 1.22]	0.145	/	
Protein intake at day 35, 0.5 g/kg/day	0.59 [0.35; 0.99]	0.049	0.94 [0.34; 2.58]	0.898
Energy intake at day 7, 10 kcal/kg/day	0.88 [0.77; 1.02]	0.081	/	
Energy intake at day 35, 10 kcal/kg/day	0.90 [0.83; 0.97]	0.009	0.92 [0.79; 1.07]	0.274
Postnatal steroids	1.90 [1.18; 3.05]	0.008	2.64 [1.49; 4.68]	< 0.001
Parenteral nutrition duration, day	1.03[1.01; 1.05]	0.012	1.02 [1.00; 1.05]	0.074
Preterm formula, 25% total enteral intake	0.77 [0.62; 0.96]	0.023	0.76 [0.59; 0.98]	0.036

*OR *odds ratio, *95% CI *95% confidence interval

*excluded from the multivariable model because of strong collinearity with small for gestational age

**Fig. 5:**
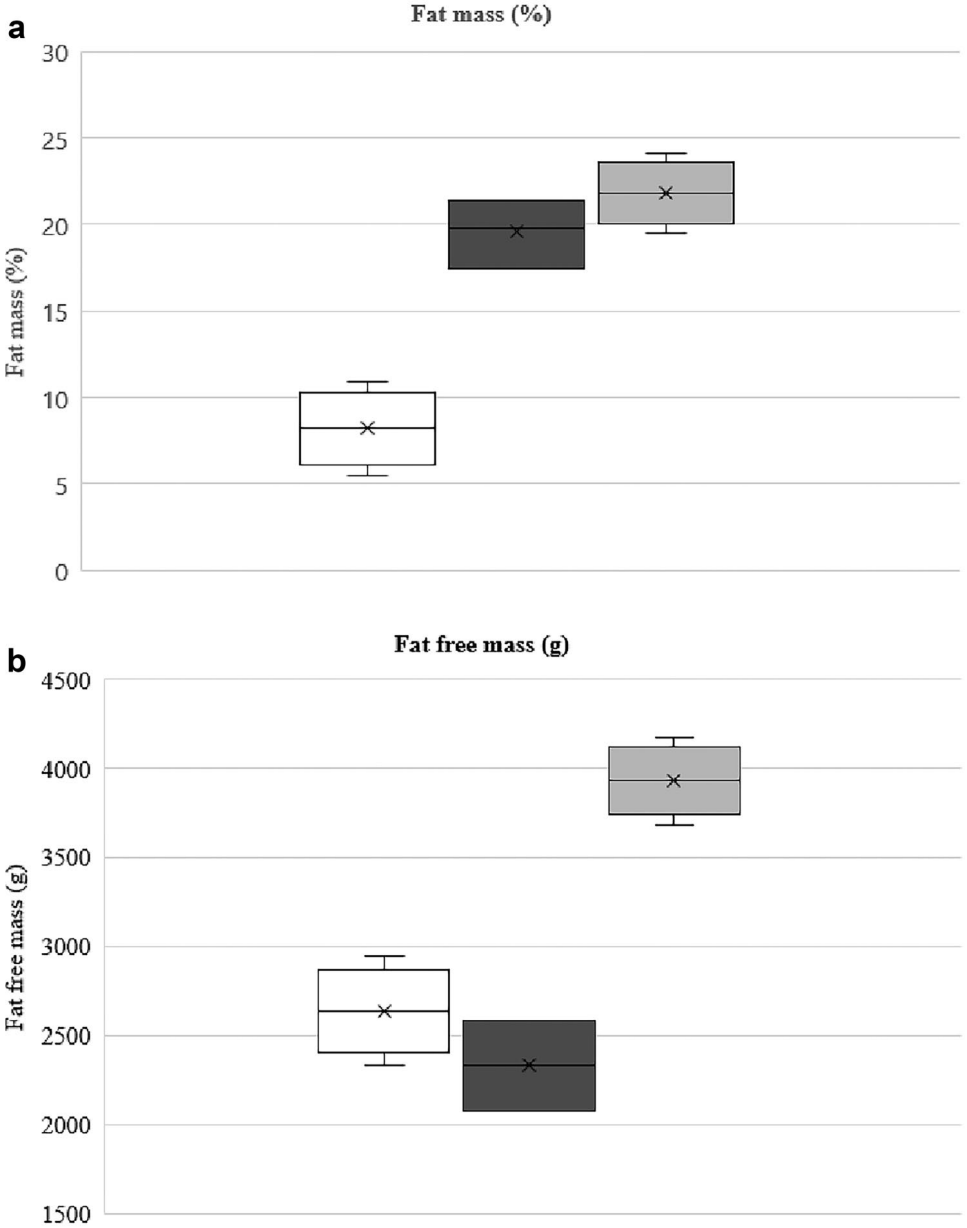
Fat mass (%) and fat-free mass (g) at discharge in 112 extremely low birth weight infants of the present cohort (dark black) compared to term infants at birth (white) and term infants at 2 months of age (grey). Reference values for term infants were obtained from ref. 10

Body composition assessment could not be performed in 198 infants, mainly because of ventilatory support **(**Fig. 1). The infants who underwent a body composition assessment had similar characteristics to the others, except for their birth weight, which was significantly higher. They were also less sick and had less PNGF (Table 5). Measurement of body composition was performed at a mean of 67 ± 15 days of life, i.e., 38 ± 1 weeks PMA. FM% was 19.8 ± 3.6% of body weight, and FFM was 2314 ± 389 g (Fig. 5). FM% tended to be lower in infants that were SGA at birth than in non-SGA infants (18.8 ± 3.6% vs. 20.1 ± 3.4% *p* = 0.093). FM% was significantly higher in infants with optimal growth compared to those with PNGF (20.2 ± 3.2% vs. 18.1 ± 4.5%, *p* = 0.036). Factors influencing FM% were the proportion of milk intake as preterm formula and GA at birth. Those influencing FFM were GA at birth and sex (Table 6).

**Table 5:** Characteristics of extremely low birth weight infants with or without PEA POD®

**Characteristics**	**With PEA POD®** ***N*= 112**	**Without PEA POD®** ***N*= 198**	***p***
Antenatal steroids, *n* (%)	110 (98)	190 (96)	0.496
Gestational age at birth, weeks	27.9 (± 1.8)	26.6 (± 1.8)	0.096
Male sex, *n* (%)	45 (40)	95 (48)	0.184
Birth weight, g	830 (± 116)	783 (± 132)	0.003
Birth length, cm	33.9 (± 2)	32.8 (± 2.2)	1.118
Birth head circumference, cm	23.9 (± 1.5)	23.6 (± 1.5)	0.104
Small for gestational age, *n* (%)	31 (28)	73 (37)	0.1
Postnatal steroids, *n* (%)	17 (15)	100 (51)	< 0.001
Bronchopulmonary dysplasia, *n* (%)	64 (57)	164 (83)	< 0.001
Periventricular leukomalacia, *n* (%)	0 (0)	5 (3)	0.163
Intraventricular haemorrhage stage 3 and 4, *n* (%)	4 (4)	12 (6)	0.341
Retinopathy of prematurity stage ≥ 3, *n* (%)	5 (5)	5 (3)	0.505
Necrotising enterocolitis stage ≥ 2, *n* (%)	4 (2)	0 (0)	0.3
Parenteral nutrition duration, days	17 (± 9)	20 (± 13)	0.128
Δ*Z*-score weight between discharge and birth	−0.15 (± 0.75)	−0.41 (± 0.72)	0.003
Postnatal growth failure, *n* (%)	15 (13)	69 (35)	< 0.001

**Table 6:** Factors associated with fat mass percentage (a) and fat-free mass (b) at discharge in 112 extremely low birth weight infants. Results are expressed as estimates with their 95% confidence interval

a.				
**Factors**	**Univariable**	***P***	**Multivariable**	***P***
Gestational age at birth, weeks	−0.89 [−1.3; −0.5]	< 0.001	−0.87 [−1.3; −0.45]	< 0.001
Sex (female vs. male)	0.34 [−1.0; 1.7]	0.623	/	
Antenatal steroids	−0.35 [−5.5; 4.8]	0.893	/	
Small for gestational age	−1.5 [−3.0; 0.1]	0.060	/	
Protein intake at day 7, 0.5 g/kg/day	0.02 [−2.20; 2.20]	0.986	/	
Protein intake at day 35, 0.5 g/kg/day	−1.70 [−3.10; −0.19]	0.027	−0.61 [−2.00; 0.77]	0.382
Energy intake at day 7, 10 kcal/kg/day	0.11 [−0.29; 0.51]	0.591	/	
Energy intake at day 35, 10 kcal/kg/day	−0.01 [−0.25; 0.23]	0.937	/	
Postnatal steroids	2.7 [0.86; 4.5]	0.004	1.49 [−0.13; 3.10]	0.071
Parenteral nutrition duration, day	0.09[0.02; 0.17]	0.015	0.03 [−0.04; 0.09]	0.361
Preterm formula, 25% total enteral intake	0.45 [0.45; 1.60]	< 0.001	1.11 [0.60; 1.6]	< 0.001

b.				
**Factors**	**Univariable**	***P***	**Multivariable**	***P***
Gestational age at birth, weeks	−130 [−166; −94]	< 0.001	−78 [−127; −0.29]	0.002
Sex (female vs. male)	−185 [−330; −40]	0.013	−168 [−294; −42]	0.009
Antenatal steroids	−214 [−765; 336]	0.442	/	
Small for gestational age	−370 [−522; −219]	< 0.001	−140 [−316; 36]	0.118
Protein intake at day 7, 0.5 g/kg/day	−39 [−82; 3.10]	0.069	/	
Protein intake at day 35, 0.5 g/kg/day	−182 [−345; −20]	0.028	−65 [−215; 84]	0.387
Energy intake at day 7, 10 kcal/kg/day	0.11 [−0.29; 0.51]	0.591	/	
Energy intake at day 35, 10 kcal/kg/day	−14 [−40; 13]	0.310	/	
Postnatal steroids	232 [33; 431]	0.023	−11 [−191; 170]	0.907
Parenteral nutrition duration, day	9.3 [1.0; 18]	0.028	2.29 [−5.9; 9.8]	0.544
Preterm formula, 25% total enteral intake	41 [−25; 107]	0.222	/	

Results are expressed as estimates with their 95% confidence intervals

## Discussion

In the present cohort of very high-risk ELBW infants, the individualised nutritional care approach applied prevented postnatal weight loss in most infants, limited length deficit, and supported excellent HC growth.

Protein intakes were close to the recommended intakes [15]. The recommended total energy intake, which may be difficult to achieve in such extremely immature infants, was reached faster than previously reported [15, 18]. Although the protein-to-energy ratio was slightly lower than recommended due to the high energy intake, the former remained higher than previously reported [19]. The high energy intake observed is likely due to the fact that the energy supplementation required after cessation of parenteral nutrition was not stopped as soon as recommended, reflecting the difficulties in fully adhering to protocols in clinical practice. However, such intakes supported early postnatal growth, as only a fifth of infants were SGA at 1 month of life, compared to the 75% previously reported, representing a significant improvement in the prevention of initial growth deficit originally described by Embleton et al. [18, 20]. Good protein utilisation was reflected by rather low serum urea. These results suggest that the slightly excessive energy intake relative to the protein intake avoided the restriction of protein utilisation which could be related to a lack of energy. Although such intakes allowed good postnatal growth in most infants, they also likely favoured high FM% at discharge. These data advocate for close monitoring of protein and energy intakes, but also that of the protein-to-energy ratio.

The postnatal growth observed in this cohort closely followed that of foetal growth, at least for body weight and HC. The present individualised nutritional care approach helped to avoid the postnatal weight deficit as demonstrated by a *Z*-score loss much lower than previously reported (−1), despite the fact that the infants herein were less mature [20]. This deficit was also lower than that reported more recently by Cormack et al. (−0.48) in a similar population and even lower than the −0.7 to −1 *Z*-score loss recently proposed as acceptable [11, 21]. In the present cohort, there were four times less infants with a *Z*-score for body weight below −2 at discharge than in the EXPRESS cohort [6]. Moreover, less than a third of infants herein had a *Z*-score for body weight below −1.28 at discharge, which is lower than previously reported in even more mature very low birth weight infants [23]. The observed absence of postnatal HC deficit in the majority of infants herein is also noteworthy, as such a deficit has been associated with suboptimal neurological outcomes [1, 24]. In a similar population, Cormack et al. reported a higher *Z*-score loss of 0.82 [22]. In the EPICure cohort, ELBW infants with a significant deficit in postnatal HC growth had HC below reference values as adults [25]. Although there is no strong evidence supporting that having an HC close to the mean for GA at discharge is associated with better neurodevelopment, it seems rather reassuring for the future of these high-risk infants. The length deficit observed herein was similar to the −1.5 and −1.16 previously reported [21, 26]. Surprisingly, length data are quite rarely reported in studies assessing postnatal growth in ELBW infants [20, 27], and very few authors reported an improvement in the *Z*-score for length during hospitalisation [28, 29]. It is well known that the final height of premature infants is approximately 1SD lower than that of term infants [30]. Furthermore, since postnatal length growth deficit can be associated with long-term consequences such as osteoporosis, large deficits in length should be avoided as much as possible [31]. The few studies that found such positive length kinetics underlined the central role of protein intake [28, 29]. This represents another reason for optimising protein intakes and protein-to-energy ratio. In summary, and in contrary to previously reported studies, the individualised nutritional care approach used in the present cohort helped limit postnatal growth deficits [32–34].

Of note, PNGF might be a more relevant marker of growth deficit than just SGA at discharge [35]. When using PNGF, the postnatal growth deficits in weight, length, and HC were present in more infants than when using SGA at discharge. Neonatologists should aim to reduce the risk of PNGF rather than SGA at discharge. This study confirmed that SGA at birth and postnatal steroids are independent risk factors for PNGF and found that the proportion of milk ingested as the preterm formula was a significant protective factor of PNGF. This could be due to the fact that preterm formula, which is used to supplement or replace absent or insufficient breast milk provides, a more stable nutritional supply than fortified breast milk.

Currently, there is no consensus regarding the body composition objective at the end of hospitalisation. Due to the metabolic adaptation to extrauterine life needed to increase energy storage and improve thermoregulation, the foetal body composition cannot serve as a reference [36]. Given that full-term neonates have an FM% of around 10% at birth and 25% at 2–3 months of life [11, 37] and that the FM% at discharge in the present ELBW cohort was 20%, the objective could be between that of a 36–40 week foetus and that of a full-term infant aged 2–3 months. This relatively high FM% at discharge is similar to that observed in smaller cohorts with similar postnatal weight change but higher than the 15% reported in less immature infants with a more favourable postnatal weight change [37, 38]. Contrary to what has been reported, the results herein showed that each additional GA week at birth resulted in a decrease in FM% [39]. Thus, the more immature the infant, the higher the FM% at discharge, which could reflect an increase in fat storage due to the difficulty in maintaining well-balanced protein and energy intakes throughout hospitalisation. Nevertheless, FM% has been shown to normalise within a few months after discharge [10]. Such a transient excess in FM% could thus be useful for ELBW infants, as it may represent the “price to pay” to avoid postnatal growth deficits, particularly regarding HC.

A deficit in FFM at discharge has been associated with neurological impairment at 2 years of age [9, 40]. Herein, FFM was lower than in term infants (2.8–2.9 kg), confirming the data published by Hamatschek et al. (mean of 2.5 kg), but was 300 g higher than that reported in a very low birth weight cohort [10, 39]. However, as the FFM is expressed in absolute value, it depends directly on body weight, and it is therefore difficult to compare studies, in which nutritional care and body weight at discharge varies greatly.

A limitation of this study is its single-centre design, although this did not prevent the data from a significant number of ELBW infants to be analysed. Furthermore, it avoided the impact of potential inter-centre differences in practices other than nutritional management, which could impact postnatal growth. Moreover, only a subgroup of infants could benefit from the body composition assessment herein. They had less bronchopulmonary dysplasia and therefore, less postnatal steroid treatment. However, even though the most severely ill infants did not undergo a body composition measurement, those who had it still represented a population of very high-risk ELBW infants.

In conclusion, an individualised nutritional care approach using standardised fortification followed by adjustable fortification limited body weight and HC postnatal growth deficits. FM% was higher than that of foetuses of the same GA, possibly representing a necessary adaptation to extrauterine life. Further studies are still needed to determine the growth and body composition objectives in ELBW infants according to their impact on later development.

## Data Availability

Data are available upon request to the corresponding author.

## Abbreviations

ELBW: Extremely low birth weight
FFM: Fat-free mass
FM: Fat mass
GA: Gestational age
HC: Head circumference
PNGF: Postnatal growth failure
PMA: Postmenstrual age
SGA: Small for gestational age
